# Tea Seed Oil Prevents Obesity, Reduces Physical Fatigue, and Improves Exercise Performance in High-Fat-Diet-Induced Obese Ovariectomized Mice

**DOI:** 10.3390/molecules24050980

**Published:** 2019-03-11

**Authors:** Yu-Tang Tung, Yi-Ju Hsu, Yi-Wen Chien, Chi-Chang Huang, Wen-Ching Huang, Wan-Chun Chiu

**Affiliations:** 1 Graduate Institute of Metabolism and Obesity Sciences, Taipei Medical University, Taipei 11031, Taiwan; f91625059@tmu.edu.tw (Y.-T.T.); ychien@tmu.edu.tw (Y.-W.C.); john5523@ntsu.edu.tw (C.-C.H.); 2 Nutrition Research Center, Taipei Medical University Hospital, Taipei City 11031, Taiwan; 3 Graduate Institute of Sports Science, National Taiwan Sport University, Taoyuan 33301, Taiwan; 1041302@ntsu.edu.tw; 4 School of Nutrition and Health Sciences, Taipei Medical University, Taipei 11031, Taiwan; 5 Research Center of Geriatric Nutrition, College of Nutrition, Taipei Medical University, Taipei 11031, Taiwan; 6 Department of Exercise and Health Science, National Taipei University of Nursing and Health Sciences, Taipei 11219, Taiwan

**Keywords:** menopause, tea seed oil, exercise performance, polyunsaturated fatty acids, monounsaturated fatty acids, saturated fatty acids

## Abstract

Menopause is associated with changes in body composition (a decline in lean body mass and an increase in total fat mass), leading to an increased risk of metabolic syndrome, nonalcoholic fatty liver disease, and heart disease. A healthy diet to control body weight is an effective strategy for preventing and treating menopause-related metabolic syndromes. In the present study, we investigated the effect of long-term feeding of edible oils (soybean oil (SO), tea seed oil (TO), and lard oil (LO)) on female ovariectomized (OVX) mice. SO, TO, and LO comprise mainly polyunsaturated fatty acids (PUFA), monounsaturated fatty acids (MUFA), and saturated fatty acids (SFA), respectively. However, there have been quite limited studies to investigate the effects of different fatty acids (PUFA, MUFA, and SFA) on physiological adaption and metabolic homeostasis in a menopausal population. In this study, 7-week-old female Institute of Cancer Research (ICR) mice underwent either bilateral laparotomy (sham group, *n* = 8) or bilateral oophorectomy (OVX groups, *n* = 24). The OVX mice given a high-fat diet (HFD) were randomly divided into three groups: OVX+SO, OVX+TO, and OVX+LO. An HFD rich in SO, TO, or LO was given to the OVX mice for 12 weeks. Our findings revealed that the body weight and relative tissues of UFP (uterus fatty peripheral) and total fat (TF) were significantly decreased in the OVX+TO group compared with those in the OVX+SO and OVX+LO groups. However, no significant difference in body weight or in the relative tissues of UFP and TF was noted among the OVX+SO and OVX+LO groups. Furthermore, mice given an HFD rich in TO exhibited significantly decreased accumulation of liver lipid droplets and adipocyte sizes of UFP and brown adipose tissue (BAT) compared with those given an HFD rich in SO or LO. Moreover, replacing SO or LO with TO significantly increased oral glucose tolerance. Additionally, TO improved endurance performance and exhibited antifatigue activity by lowering ammonia, blood urea nitrogen, and creatine kinase levels. Thus, tea seed oil (TO) rich in MUFA could prevent obesity, reduce physical fatigue, and improve exercise performance compared with either SO (PUFA)- or LO(SFA)-rich diets in this HFD-induced obese OVX mice model.

## 1. Introduction

Menopause is usually associated with an increase in body weight and body fat accumulation in the waist region, leading to an increased risk of metabolic syndrome, nonalcoholic fatty liver disease (NAFLD), and heart disease [1,2,3,4]. Premenopausal women exhibit a lower risk of cardiovascular disease than men with the same body mass index, which may be because estrogen prevents liver fat accumulation and stimulates fat oxidation, thereby preventing insulin resistance [5,6,7,8,9,10,11]. Recent studies have demonstrated that mice with estrogen receptor alpha (ERα) mutations, ovariectomized (OVX) rodents, mice with global ERα knockout, and mice lacking aromatase may undergo loss of estrogen signaling, thereby leading to an increase in liver fat [12,13,14,15,16]. Mittendorfer et al. showed that estrogen deficiency leads to redistribution of body fat and accumulation of visceral fat, which may affect NAFLD development and progression [7]. Exogenous estrogen was demonstrated to prevent many menopause-related metabolic abnormalities; however, long-term hormone replacement therapy may increase the risk of breast cancer [17]. Lifestyle changes, including healthy diets and regular exercise to reduce body weight, are the strategies for preventing and treating menopause-related metabolic syndromes [18,19].

Tea seed oil (TO) is an edible oil obtained by squeezing mature seeds of tea (*Camellia oleifera*), and its fatty acid composition is similar to that of olive oil. Its monounsaturated fatty acid (MUFA) content is higher than that of olive oil, and its unsaturated fatty acid content fully complies with international nutritional standards for the Omega diet [20]. Besides this, a previous study demonstrated that hydroxytyrosol and tyrosol in olive oil have remarkable antioxidative and anti-inflammatory effects against reactive oxygen species (ROS)-induced damage [21]. Tea seed oil contains multifarious functional ingredients such as saponins, polyphenols, vitamin E, squalene, and flavonoids [22]. Tea seed oil, like olive oil, also has antioxidative ingredients. Long-term intake of TO can considerably lower blood pressure, lower blood lipids, delay atherosclerosis, prevent cardiovascular sclerosis, increase gastrointestinal absorption function, promote hormone secretion of the endocrine glands, prevent a decline in neurological function, improve human immunity, and prevent cancer [23]. 

Metabolic changes caused by estrogen depletion in OVX rodents have many characteristics similar to changes in menopausal women, including weight gain, increased obesity, adipose tissue inflammation, and fatty liver inflammation [24,25]. These similarities make OVX mice suitable models for studying postmenopausal physiological changes. Therefore, in the present study, we investigated the effect of the following three dietary oils on female OVX mice: soybean oil (SO), which contains 24% oleic acid and 54% linoleic acid; TO, which mainly contains 78% oleic acid (C18:1), 9% linoleic acid (C18:2), 0.4% linolenic acid (C18:3), 9% palmitic acid (C16:0), and 2.0% stearic acid (C18:0); and lard oil (LO), which contains 24% palmitic acid (16:0) and 44% oleic acid (18:1). SO, TO, and LO comprise mainly polyunsaturated fatty acids (PUFA), monounsaturated fatty acids (MUFA), and saturated fatty acids (SFA), respectively. Menopausal symptoms generally include hot flashes, night sweat, sleep problems, anxiety, depression, hormone changes, and fatigue. In clinical survey studies, 86% of Japanese menopausal women felt tiredness more than once a week, and even 49% felt it almost every day [26]. The postmenopausal women’s exercise tolerance could decrease, associated with the loss of potent estrogens, insulin resistance, and endothelial dysfunction [27]. In addition, declines in physical activity and muscle strength were demonstrated not only in menopausal women [28] but also in OVX and estrogen receptor α (ERα) knockout animal models [29]. In previous studies, nutrient supplementation, such as with soy lecithin and estradiol, resulted in fatigue alleviation and physical activity improvement [30,31]. 

However, there have been quite limited studies to investigate the effects of different fatty acids (PUFA, MUFA, and SFA) on physiological adaption and metabolic homeostasis in a menopausal population. Therefore, we hypothesized that the long-term consumption of the different fatty acids would exert beneficial effects not only on obesity and glucose regulation but also on exercise performance using an ovariectomy and high-fat diet (HFD)-induced obese mouse model.

## 2. Results

### 2.1. Effects of SO, TO, and LO on Food Intake, Body Weight, and Tissue Weights of OVX and HFD-Induced Obese Mice

**Figure 1:**
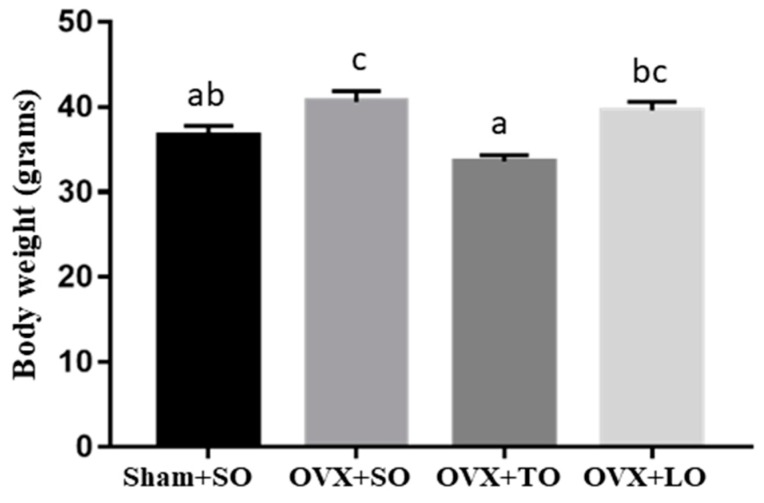
Effect of soybean oil (SO), tea oil (TO), and lard oil (LO) on body weight in ovariectomized and high-fat-diet-induced obese mice at the end of the experiment. Data are presented as mean ± SEM, *n* = 8 mice/group. Bars with different letters (a, b, c) indicate a significant difference at *p* < 0.05 determined using one-way ANOVA.

No significant difference in food intake was observed among the Sham+SO (3.10 ± 0.06 g), OVX+SO (2.92 ± 0.06 g), and OVX+TO (2.91 ± 0.06 g) groups. However, the food intake of the OVX+LO group (2.88 ± 0.10 g) was significantly lower than those of the other groups (*p* < 0.05). The initial body weights were 32.9 ± 0.08, 33.9 ± 0.08, 33.7 ± 0.08, and 33.8 ± 0.07 g in the Sham+SO, OVX+SO, OVX+TO, and OVX+LO groups, respectively, and it did not significantly differ among the groups (F(3, 28) = 0.345, *p* = 0.786, η^2^ = 0.137). After supplementation and high-fat diet induction (Figure 1), a significant difference was observed among groups (F(3, 28) = 8.956, *p* = 0.001, η^2^ = 0.576), and body weight gain was significantly higher in the OVX+SO group than in the Sham+SO group (*p* < 0.05). However, the body weight in the OVX+TO group was significantly decreased compared with that in the OVX+SO and OVX+LO groups (*p* < 0.05). As shown in Table 1, the relative tissue weights (liver, heart, lung, and brown adipose tissue (BAT)) of the mice in the OVX+SO group were significantly lower than those of the Sham+SO group. Also, uterus fatty peripheral (UFP) and total fat (TF) contents in the OVX+SO group were higher than those in the Sham+SO group (*p* < 0.05). However, the relative tissue weights of the liver, heart, lung, muscle, and BAT mass were significantly elevated in the OVX+TO group, whereas the relative tissue weights of UPF and TF were decreased compared with these weights in the OVX+SO and OVX+LO groups. 

### 2.2. Effects of SO, TO, and LO on Exhaustive Swimming Time and Forelimb Grip Strength in OVX and HFD-Induced Obese Mice

**Figure 2:**
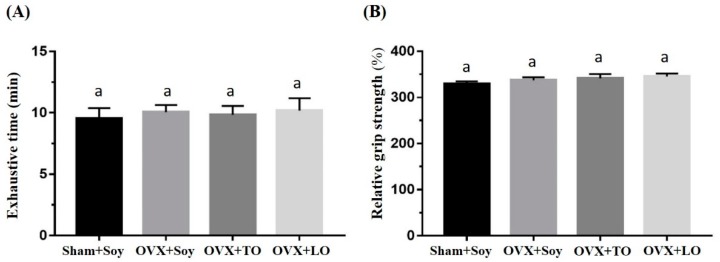
Effects of soybean oil (SO), tea seed oil (TO), and lard oil (LO) on (**A**) the exhaustive swimming time and (**B**) forelimb grip strength in ovariectomized and high-fat-diet-induced obese mice at the beginning of the experiment. The exhaustive swimming and grip strength were assessed before the supplementation and induction. Data are presented as mean ± SEM, *n* = 8 mice/group. Bars with different letters (a, b) indicate a significant difference at *p* < 0.05 determined using one-way ANOVA.

**Figure 3:**
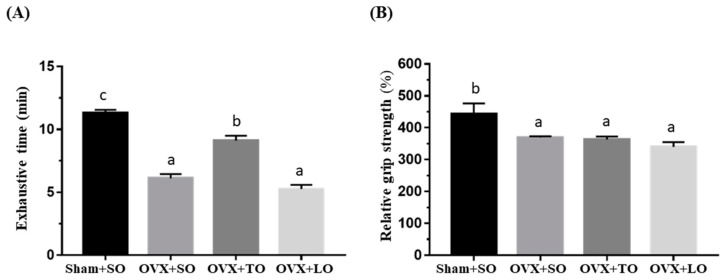
Effects of soybean oil (SO), tea seed oil (TO), and lard oil (LO) on (**A**) the exhaustive swimming time and (**B**) forelimb grip strength in ovariectomized and high-fat-diet-induced obese mice at the end of the experiment. After 12 weeks of intervention, exhaustive swimming and grip strength were assessed. Data are presented as mean ± SEM, *n* = 8 mice/group. Bars with different letters (a, b, c) indicate a significant difference at *p* < 0.05 determined using one-way ANOVA.

The basal levels of physical activities before the supplementation and induction are illustrated in Figure 2, and there were no significant differences in exhaustive swimming endurance and grip strength among the groups (F(3, 28) = 0.115, *p* = 0.95, η^2^ = 0.112 and F(3, 28) = 0.956, *p* = 0.432, η^2^ = 0.194, respectively). However, the exhaustive swimming endurance and grip strength were significantly different among the groups (F(3, 28) = 67.25, *p* < 0.0001, η^2^ = 0.910 and F(3, 28) = 5.619, *p* = 0.006, η^2^ = 0.457, respectively) at the end of experiment (Figure 3), and the ovariectomy resulted in significant reductions in the exhaustive swimming time and forelimb grip strength (*p* < 0.05). The exhaustive swimming time was longer in the OVX+TO group than in the OVX+SO and OVX+LO groups (*p* < 0.0001). Thus, the exhaustive swimming time in the OVX+TO group was significantly higher by 49% and 72% than those in the OVX+SO and OVX+LO groups, respectively. Therefore, replacing SO and LO with TO can lengthen the exhaustive swimming time in HFD-induced obese OVX mice. In terms of the grip strength, there was no significant difference among the three OVX groups, but the SO, TO, and LO treatments showed a significant decrease by 16–23% as compared to the Sham+SO group (*p* = 0.045, 0.032, and 0.005, respectively).

### 2.3. Effects of SO, TO, and LO on Lactate, Ammonia, BUN, and CK Levels After the 10 Minutes Swimming Test in OVX and HFD-Induced Obese Mice

**Figure 4:**
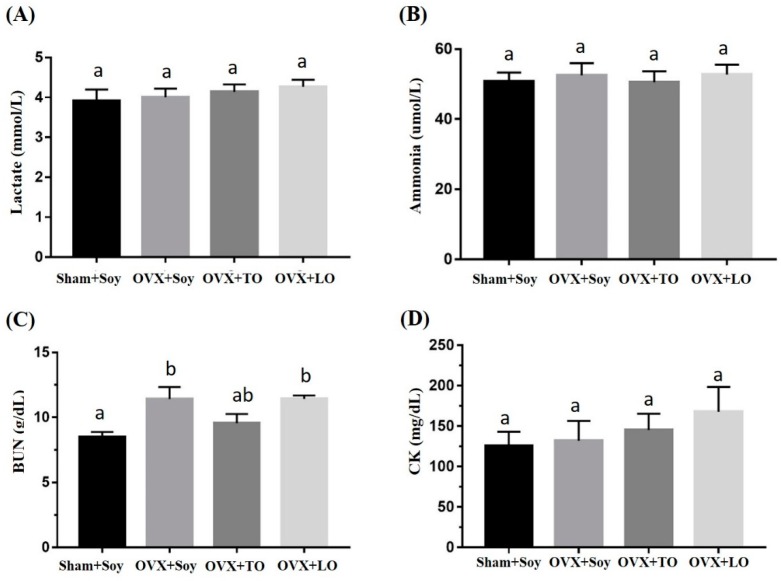
Effects of soybean oil (SO), tea seed oil (TO), and lard oil (LO) on (**A**) lactate, (**B**) ammonia, (**C**) BUN, and (**D**) CK levels in ovariectomized and high-fat-diet-induced obese mice. After 12 weeks of intervention, fatigue-associated biochemical indices were assessed before a swimming test. Data are presented as mean ± SEM, *n* = 8 mice/group. Bars with different letters (a, b) indicate a significant difference at *p* < 0.05 determined using one-way ANOVA.

**Figure 5:**
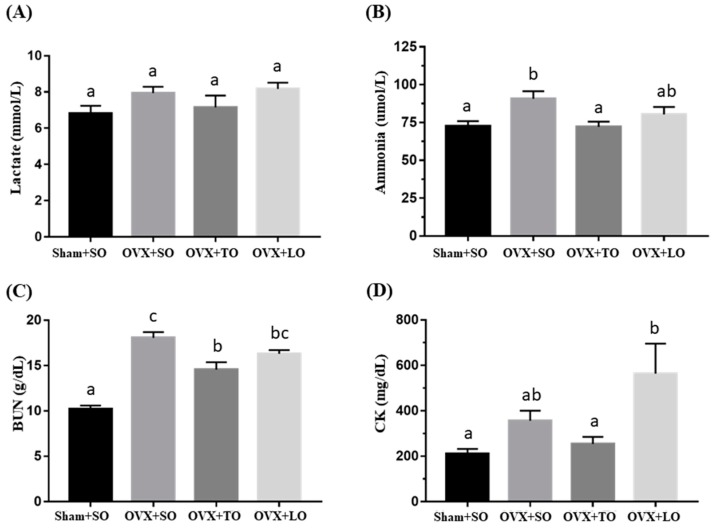
Effects of soybean oil (SO), tea seed oil (TO), and lard oil (LO) on (**A**) lactate, (**B**) ammonia, (**C**) BUN, and (**D**) CK levels in ovariectomized and high-fat-diet-induced obese mice. After 12 weeks of intervention, fatigue-associated biochemical indices were assessed after a 10 min swimming test. Data are presented as mean ± SEM, *n* = 8 mice/group. Bars with different letters (a, b, c) indicate a significant difference at *p* < 0.05 determined using one-way ANOVA.

The effects of SO, TO, and LO on lactate, ammonia, BUN (Blood Urea Nitrogen), and CK (Creatine Kinase) levels after 10 min swimming test are shown in Figure 4 and Figure 5. The basal levels of lactate, ammonia, and BUN are illustrated in Figure 4, and no significant difference among the groups in the indicated indices were observed before the swimming test (F(3, 28) = 0.481, *p* = 0.698, η^2^ = 0.049; F(3, 28) = 0.151, *p* = 0.928, η^2^=0.016; and F(3, 28) = 0.606, *p* = 0.616, η^2^ = 0.061) except for the level of CK (F(3, 28) = 5.347, *p* = 0.007, η^2^ = 0.445). After swimming, the ammonia, BUN, and CK levels demonstrated significant differences among groups (F(3, 28) = 4.179, *p* = 0.019, η^2^ = 0.485; F(3, 28) = 31.858, *p* < 0.0001, η^2^ = 0.872; and F(3, 28) = 4.774, *p* = 0.011, η^2^ = 0.417, respectively) but not in the lactate (F(3, 28) = 1.912, *p* = 0.16, η^2^ = 0.223) (Figure 5). The ammonia and BUN levels in the OVX+SO group were significantly higher by 25% (*p* = 0.032) and 78% (*p* < 0.0001), respectively, than those in the Sham+SO group. However, the ammonia and BUN levels in the OVX+TO group were significantly lower (21%, *p* = 0.027; 19%, *p* = 0.03, respectively) than those in the OVX+SO group. Moreover, no significant differences in lactate, ammonia, and BUN levels were noted between the OVX+SO and OVX+LO groups (*p* = 0.984, 0.337, and 0.196, respectively). However, the CK levels in the OVX+LO group were significantly higher (58%, *p* = 0.012) than those in the Sham+SO group. Therefore, replacing SO and LO with TO can reduce the ammonia and BUN levels at 10 min after a swimming test in HFD-induced obese OVX mice.

### 2.4. Effects of SO, TO, and LO on Liver Glycogen in OVX and HFD-Induced Obese Mice

**Figure 6:**
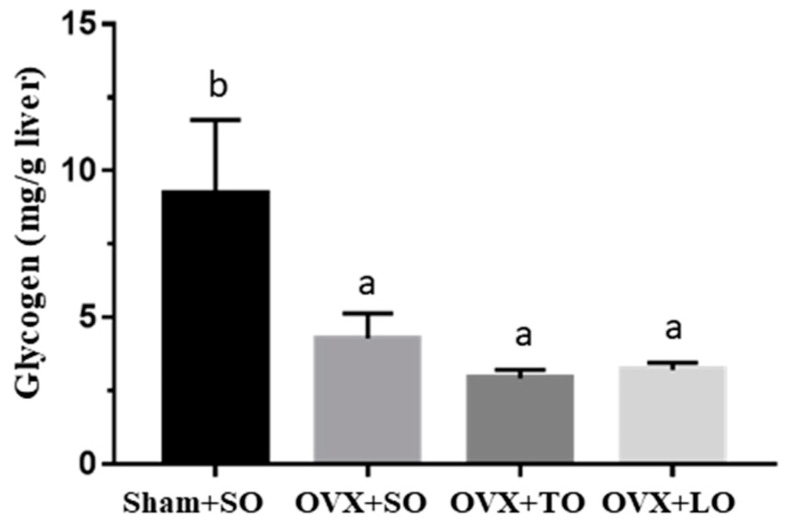
Effects of soybean oil (SO), tea seed oil (TO), and lard oil (LO) on liver glycogen content in ovariectomized and high-fat-diet-induced obese mice. Data are presented as mean ± SEM, *n* = 8 mice/group. Bars with different letters (a, b) indicate a significant difference at *p* < 0.05 determined using one-way ANOVA.

As shown in Figure 6, the liver glycogen content exhibited significant differences among treatments (F(3, 28) = 4.9, *p* = 0.01, η^2^ = 0.524), and the levels in the OVX+SO, OVX+TO, and OVX+LO groups were lower by 54% (*p* = 0.042), 68% (*p* = 0.015), and 65% (*p* = 0.021), respectively, than that in the Sham+SO group. Moreover, no significant differences in liver glycogen content was noted among the OVX+SO, OVX+TO, and OVX+LO groups.

### 2.5. Effects of SO, TO, and LO on Oral Glucose Tolerance and AUC in OVX and HFD-Induced Obese Mice

**Figure 7:**
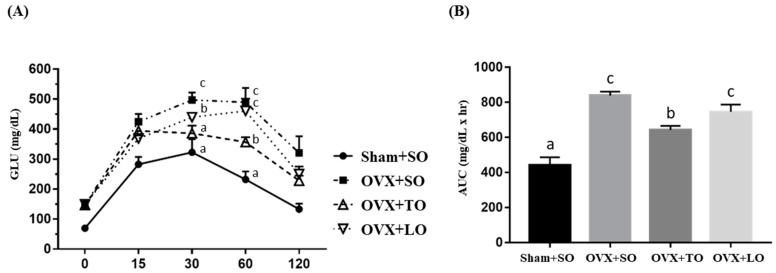
Effects of soybean oil (SO), tea seed oil (TO), and lard oil (LO) on (**A**) oral glucose tolerance and (**B**) area under the curve (AUC) in ovariectomized and high-fat-diet-induced obese mice. After 12 weeks of intervention, blood was collected from the caudal artery at the indicated time points for glucose content assessment. Data are presented as mean ± SEM, *n* = 8 mice/group. Bars with different letters (a, b, c) indicate a significant difference at *p* < 0.05 determined using one-way ANOVA.

The effects of SO, TO, and LO on oral glucose tolerance and area under the curve (AUC) in OVX mice are shown in Figure 7A,B, respectively. The OVX+SO group exhibited significantly decreased oral glucose tolerance and increased AUC compared with the Sham+SO group. In the glucose tolerance test, shown in Figure 7A, a significant difference was observed for the main effects of both treatment and time (F(3, 28) = 27.1, *p* < 0.0001, and F(4, 88) = 87.4, *p* < 0.0001, respectively). However, the OVX+TO group exhibited significantly increased oral glucose tolerance when compared with the OVX+SO group by Tukey post-hoc analysis (*p* = 0.0001). In addition, the interaction effect (treatment × time) also demonstrated a significant difference (F(12, 88) = 1.88, *p* = 0.049). Additionally, there was a significant difference among groups in terms of AUC index (F(3, 28) = 24.02, *p* < 0.0001, η^2^ = 0.783) (Figure 7B), and the Sham+SO group exhibited a significantly lower AUC than did the OVX+SO (*p* < 0.0001), OVX+TO (*p* = 0.003), and OVX+LO (*p* < 0.0001) groups. Among the OVX treatments, the OVX+TO group exhibited the smallest AUC compared to the OVX+SO (*p* = 0.004) and OVX+LO (*p* = 0.042) groups.

### 2.6. Effects of SO, TO, and LO on Biochemical Variables in OVX and HFD-Induced Obese Mice at the End of the Experiment

The biochemical indices evaluated, namely, AST (aspartate transaminase), ALT (alanine transaminase), TC (Total cholesterol), TG (Triglyceride), BUN, creatinine, ALB (Albumin), TP (Total protein), and LDH (Lactate Dehydrogenase), are shown in Table 2. The TG, BUN, and creatinine levels in the OVX+SO group were significantly higher than those in the Sham+SO group (F(3, 28) = 9.06, *p* = 0.001, η^2^ = 0.576; F(3, 28) = 5.347, *p* = 0.007, η^2^ = 0.445; and F(3, 28) = 3.29, *p* = 0.042, η^2^ = 0.33, respectively) and the ALB level in the OVX treatment groups was significantly lower than that in the Sham+SO group (F(3, 28) = 5.99, *p* = 0.004, η^2^ = 0.473). Other biochemical indices, namely insulin, AST, ALT, TC, TP, and LDH, did not significantly differ among the four groups. However, the BUN and creatinine levels in the OVX+TO group were slightly lower (16%, *p* > 0.05; 11%, *p* > 0.05, respectively) than that in the OVX+SO group. Notably, the TG level in the OVX+LO group was significantly higher than those in the OVX+SO and OVX+TO groups.

### 2.7. Effects of SO, TO, and LO on Histopathology of Tissues at the End of the Experiment in OVX and HFD-Induced Obese Mice

**Figure 8:**
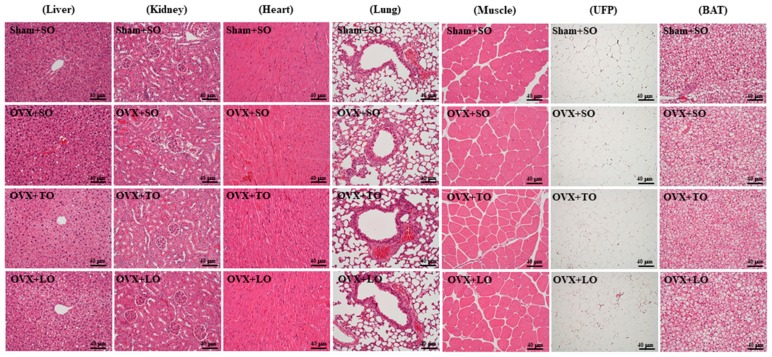
Effects of soybean oil (SO), tea seed oil (TO), and lard oil (LO) on the liver, kidney, heart, lung, muscle, UFP, and BAT tissues in ovariectomized and high-fat-diet-induced obese mice. Specimens were observed using light microscopy. Hematoxylin and eosin stain, magnification: ×200.

The pathological histologies of tissues, namely the liver, kidney, heart, lung, muscle, UFP, and BAT tissues, are shown in Figure 8. The histological observations of the kidney, heart, lung, and muscle of the mice in the OVX+SO, OVX+TO, and OVX+LO groups did not significantly differ from those in the Sham+SO group. The OVX mice on diet intervention with SO exhibited marked hepatomegaly and fatty liver formation compared with the sham mice on diet intervention with SO; however, the OVX mice on diet interventions with TO exhibited significant reductions in hepatomegaly and fatty liver formation compared with OVX mice on diet intervention with SO and LO. In addition, large adipocytes of UFP and BAT were markedly increased in the OVX+SO group compared with in the Sham+SO group. However, OVX mice on an HFD rich in SO or LO exhibited significantly larger adipocytes of UFP and BAT than did OVX mice on an HFD rich in TO.

## 3. Discussion

Menopause is associated with changes in body composition (a decline in lean body mass and an increase in TF mass) and rapid loss of bone minerals [31]. Kotani et al. [32] highlighted that menopause significantly accelerates visceral fat accumulation. Lovejoy et al. [33] demonstrated that with age, middle-aged women had increased subcutaneous abdominal fat, whereas menopause is associated with increased body fat and visceral fat in the abdomen, which is associated with a reduction in energy expenditure and fat oxidation. Therefore, estrogens play a vital role in adipose cell metabolism and fat distribution [26]. Dietary ingredients may play a key role in improving insulin sensitivity and reducing the risk of diabetes and its complications [34]. SO, TO, and LO comprise mainly PUFA, MUFA, and SFA, respectively. Ryan et al. [35] reported that in patients with type 2 diabetes, a change from a PUFA diet to MUFA diet reduces insulin resistance and restores endothelium-dependent vasodilatation. Mata et al. [36] demonstrated that at a high fat intake (36% as calories), a MUFA-rich diet results in less atherogenic lipids compared with either PUFA- or SFA-rich diets. TO has a higher MUFA content than olive oil, and its unsaturated fatty acid content is fully compliant with the international nutritional standards of the Omega diet. In addition, TO contains higher levels of vitamin E (twice as much as that in olive oil) as well as squalene and flavonoids [20].

The OVX mice on an HFD rich in SO consistently exhibited increased body weight compared with the sham mice fed on an HFD rich in SO. This result is in favorable agreement with that of McElroy and Wade [37], who reported that the weight gain caused by ovariectomy is due to an increase in all carcass components (especially in total carcass lipid). However, body weight was significantly decreased in the OVX+TO group compared with in the OVX+SO and OVX+LO groups. Additionally, the relative tissue weights of UFP and TF were higher in the OVX+SO group than in the Sham+SO group, and replacing SO or LO with TO reduced the relative tissue weights of UFP and TF. However, no significant difference in body weight or in the relative tissue weights of UFP and TF was observed between the OVX+SO and OVX+LO groups. Wang et al. [38] also showed that an HFD rich in LO or SO had no significant effect on the incidences of hyperphagia and obesity. Compared with the SFA-rich diet, the MUFA-rich diet demonstrated an obvious decrease in fat deposition in humans [39]. Moreover, TO can improve human enzyme activities, increase the metabolic rate, and maintain high energy levels [20]. Furthermore, compared with the sham mice that were given an HFD rich in SO, the OVX mice that were given an HFD rich in SO exhibited a marked accumulation of liver lipid droplets and increased adipocyte sizes of UFP and BAT. Estrogen deficiency is associated with liver fat accumulation in women and rodents [3,40]. However, an HFD rich in TO significantly reduced the accumulation of liver lipid droplets and adipocyte sizes of UFP and BAT when compared with an HFD rich in SO and LO.

In this study, the OVX mice on an HFD diet exhibited significantly increased serum levels of TG, BUN, and creatinine compared with the sham mice on the same HFD diet [41]. Ke et al. [42] demonstrated no differences in BUN level between sham rats and 4-month-old OVX rats but found an increase in the BUN level in the 12-month-old OVX rats compared with sham rats. Post TO supplementation, the treated group exhibited a slight reduction in serum levels of TG, BUN, and creatinine compared with the OVX+SO group. Deng et al. [43] studied the effects of TO intake on the blood lipids of healthy adults and found that consuming 40 g/day reduced the levels of TG, TC, and LDL-C by 15.9%, 9.6%, and 13%, respectively. Deng et al. [44] also showed that TO could lower both TC and TG levels in male albino Wistar rats. The OVX mice that were given an HFD rich in SO exhibited significantly decreased oral glucose tolerance compared with the sham mice fed on an HFD rich in SO. The OVX mice exhibited metabolic disorders associated with estrogen deficiency, including elevated fasting blood glucose and obesity [3,40,45]. Roger et al. demonstrated increased fasting blood glucose levels in mice 12 weeks after ovariectomy compared with sham-operated mice [24]. However, the OVX+TO group exhibited significantly increased oral glucose tolerance compared with the OVX+SO and OVX+LO groups. Wang et al. [38] reported that obese rats that were continuously given an HFD rich in LO exhibited the highest fasting serum insulin level. However, in the subgroup of overweight subjects, SFA reduced insulin sensitivity (by 24%) compared with MUFA [34].

To examine the effectiveness of TO in improving the exercise endurance capacity in ovariectomized and HFD-induced obese mice, all animals underwent a swim-to-exhaustion exercise test. Compared with the OVX+SO group, the swimming time to exhaustion was significantly prolonged in the OVX+TO group, and hence, TO significantly improved the exercise endurance of the test animals. In this study, we found that replacing SO or LO with TO for 12 weeks can reduce blood ammonia, BUN, and CK levels, which are key blood biochemical parameters related to fatigue. Moreover, TO contains a higher squalene content, and Zhao et al. [46] revealed that squalene exhibits antifatigue ability. In addition, according to the previous study, the unsaturated fatty acids in tea oil could show much stronger antioxidative activities [47]. Therefore, we found that the TO could alleviate the indices, including blood ammonia, BUN, and CK, induced by exercise oxidative stress or injury. In addition, energy utilization could also be another factor modulated by TO supplementation. In the glucose tolerance result, the TO could improve the glucose sensitivity as compared to OVX groups with other oil supplementation. Therefore, TO supplementation could enhance exercise endurance, possibly by the beneficial effects of antioxidation and energy utilization. A previous study showed that a diet rich in TO can alleviate oxidative stress and inflammatory markers in women with hypercholesterolemia [48]. TO can alleviate damage to the gastric mucosa by ethanol through reducing lipid peroxidation, apoptosis-related proteins, proinflammatory cytokines, and nitric oxide production and by increasing antioxidant enzyme activities, heat shock proteins, and prostaglandin E2 production [49]. TO can ameliorate ketoprofen-induced gastrointestinal mucosal injury through reductions in inflammation, impairment of the antioxidant system, and oxidative damage [50]. TO can improve acetic-acid-induced colitis through reducing inflammatory damage and lipid peroxidation [22]. Therefore, TO may decrease OVX and high-fat-diet-induced obesity, improve endurance performance, and exhibit antifatigue activity, which may be related to the reduction of oxidative stress and inflammatory response. We also believe that this is an important issue to bring to possible applications to human health promotion. In the current experimental design, the indicated oil was mixed with the AIN-93M recipe to replace the oil components for daily diet uptake. Therefore, we believe that daily TO should be considered as a dietary supplement for human health benefit based on the current postmenopausal animal study.

## 4. Materials and Methods

### 4.1. Preparation of Oils

Commercially available refined SO, TO, and LO were purchased from the Taiwan Sugar Corporation (Tainan, Taiwan), Pinglin District Farmer’s Association (New Taipei, Taiwan), and I-Mei Foods Company (Taipei, Taiwan), respectively. All oil samples were stored in sealed containers at 4 °C until further use.

### 4.2. Animals and Experiment Design

Female Institute of Cancer Research (ICR) mice (age: 6 weeks) purchased from BioLASCO (A Charles River Licensee Corp., Yilan, Taiwan) were kept at 24 ± 2 °C under humidity-controlled (65% ± 5%) conditions on a 12 h light/dark cycle. The mice were allowed one week to acclimatize to the environment and diet. The mice were given access to the AIN-93M diet and distilled water ad libitum. This study was approved by the Institutional Animal Care and Use Committee of National Taiwan Sport University, Taoyuan City, Taiwan (LAC-2016-0451). After Zoletil 50 (Virbac, France)/Rompun (Bayer, Germany) anesthesia was administered, the 7-week-old female ICR mice were subjected to either bilateral laparotomy (sham group, *n* = 8) or bilateral oophorectomy (OVX groups, *n* = 24). Two weeks after surgery, the OVX mice that were given an HFD were randomly divided into three groups: OVX+SO, OVX+TO, and OVX+LO. The HFD (modified AIN-93M diet) contained 245 g of cornstarch, 80 g of maltodextrin, 100 g of sucrose, 200 g of casein, 2 g of l-cystine, 275 g of oil (SO group: 275 g of SO; TO group: 40 g of SO and 235 g of TO; and LO group: 40 g of SO and 235 g of LO), 50 g of cellulose, 35 g of mineral mixture, 10 g of vitamin mixture, and 3 g of choline bitartrate per kg diet; the energy content of this diet was 4.983 kcal/g, comprising 16.1% calories from protein, 49.7% calories from fats, and 34.2% calories from carbohydrates. After 12 weeks of intervention, the exhaustive swimming, grip strength, glucose tolerance, and fatigue-associated biochemical indices were assessed. The mice were sacrificed, and samples (tissues and serum) were stored at −80 °C for further analysis. The liver, kidney, heart, lung, muscle, brown adipose tissue (BAT), uterus fatty peripheral (UFP), and total fat (TF) were collected for weight record and pathological examination. In this study, we conducted outcome evaluations blinded to the group allocation of mice to avoid the effects of personal bias.

### 4.3. Exhaustive Swimming Test

An exhaustive swimming test was conducted according to the method of Kan et al. [51]. The swimming time from the beginning until exhaustion was measured to evaluate endurance performance.

### 4.4. Forelimb Grip Strength

A low-force testing system (Model-RX-5, Aikoh Engineering, Nagoya, Japan) was used to measure the forelimb absolute grip strength, as previously described [52].

### 4.5. Fatigue-Associated Biochemical Indices

Blood samples were collected within 10 min of the swimming exercise after 12 hours of fasting. Serum was centrifuged for 10 min (1500*g*) at 4 °C. Lactate, ammonia, blood urea nitrogen (BUN), and creatine kinase (CK) levels were determined to evaluate fatigue-associated changes by using an autoanalyzer (Hitachi 7060, Hitachi, Tokyo, Japan).

### 4.6. Tissue Glycogen Determination

Following the experiments, liver glycogen content was analyzed. Glycogen was analyzed according to our previously described method [52].

### 4.7. Histological Staining of Tissues

Various tissues were collected and fixed in 10% formalin after the mice were sacrificed. Hematoxylin and eosin staining was performed according to a previously described method by Huang et al. [53].

### 4.8. Oral Gavage Glucose Tolerance Test

The mice underwent fasting for 12 h; fasting blood glucose was analyzed. Subsequently, glucose solution (2 g/kg body weight) was administered orally, and blood was successively collected at 15, 30, 60, and 120 min. The blood was collected from the caudal artery by needle puncture at the indicated serial time points for glucose level assessment. The glucose content was determined using a glucose meter (ACCU-CHEK^®^, Roche, Indianapolis, IN, USA). Glucose area under the curve (AUC) = 0.25 × (fasting value) + 0.5 × (half-hour value) + 0.75 × (1 h value) + 0.5 × (2 h value).

### 4.9. Blood Biochemical Assessments

At the end of the experiments, all mice underwent fasting for 12 h, and blood was withdrawn through cardiac puncture. Serum was collected through centrifugation, and the levels of insulin, aspartate aminotransferase (AST), alanine aminotransferase (ALT), total cholesterol (TC), triacylglycerol (TG), BUN, creatinine, albumin (ALB), total protein (TP), and lactate dehydrogenase (LDH) were assessed using an autoanalyzer (Hitachi 7060).

### 4.10. Statistical Analysis

Experimental data are expressed as mean ± SEM (*n* = 8). The parametric one-way ANOVA was employed to calculate the significant differences (physical activities, biochemical indices, and glycogen content) among groups, followed by Tukey multiple range tests, and mixed two-way ANOVA was also applied to the glucose tolerance test. The effect size was calculated as partial eta-squared (η^2^). Statistical significance was set at *p* < 0.05. 

## 5. Conclusions

In the present study, we found that replacing SO and LO with TO reduced ovariectomy-induced weight gain, improved liver lipid profiles, and alleviated metabolic syndrome in the postmenopausal condition. Also, TO can improve endurance performance and exhibits antifatigue activity by lowering ammonia, BUN, and CK levels. Thus, the intake of an HFD rich in MUFA from tea seed oil could prevent obesity, reduce physical fatigue, and improve exercise performance compared with the intake of PUFA- or SFA-rich diets in the ovariectomized female mouse model. For practical applications, MUFA, the major fatty acid component of tea seed oil, could be a beneficial alternative option for daily intake or oil replacement for the menopausal population for health promotion purposes. 

## Figures and Tables

**Table 1:** Effects of soybean oil (SO), tea seed oil (TO), and lard oil (LO) on the relative tissue weights in ovariectomized (OVX) and high-fat-diet-induced obese mice.

Characteristic	Sham+SO	OVX+SO	OVX+TO	OVX+LO
Relative liver weight (%)	4.02 ± 0.16 ^b^	3.59 ± 0.07 ^a^	4.09 ± 0.16 ^b^	3.54 ± 0.11 ^a^
Relative kidney weight (%)	1.03 ± 0.06 ^a^	0.84 ± 0.02 ^a^	0.93 ± 0.05 ^a^	0.86 ± 0.06 ^a^
Relative heart weight (%)	0.44 ± 0.02 ^b^	0.40 ± 0.01 ^a^	0.47 ± 0.01 ^b^	0.42 ± 0.02 ^a^
Relative lung weight (%)	0.56 ± 0.03 ^b^	0.51 ± 0.03 ^a^	0.64 ± 0.02 ^b^	0.54 ± 0.02 ^a^
Relative muscle weight (%)	0.89 ± 0.02 ^b,c^	0.87 ± 0.03 ^a,b^	0.98 ± 0.02 ^c^	0.83 ± 0.03 ^a^
Relative BAT weight (%)	0.23 ± 0.01 ^c^	0.18 ± 0.00 ^a^	0.22 ± 0.01 ^b^	0.19 ± 0.01 ^a^
Relative UPF weight (%)	4.31 ± 0.62 ^a^	6.07 ± 0.65 ^b^	4.76 ± 0.50 ^a,b^	5.72 ± 0.23 ^a,b^
Relative TF weight (%)	7.96 ± 0.28 ^a,b^	9.41 ± 0.64 ^b^	7.19 ± 0.32 ^a^	9.17 ± 0.12 ^b^

Data are presented as mean ± SEM, *n* = 8 mice/group. Different letters (a, b, c) in the same row indicate a significant difference at *p* < 0.05 determined using one-way ANOVA. Muscle mass includes both gastrocnemius and soleus muscles in the back part of the lower legs. BAT, brown adipose tissue; UFP, uterus fatty peripheral; TF, total fat.

**Table 2:** Effects of soybean oil (SO), tea oil (TO), and lard oil (LO) on biochemical analysis of ovariectomized and high-fat-diet-induced obese mice.

Parameter	Sham+SO	OVX+SO	OVX+TO	OVX+LO
**Insulin (μIU/mL)**	1.69 ± 0.11 ^a^	1.11 ± 0.10 ^a^	1.63 ± 0.24 ^a^	1.26 ± 0.17 ^a^
**AST (U/L)**	116 ± 4 ^a^	131 ± 8 ^a^	112 ± 7 ^a^	138 ± 11 ^a^
**ALT (U/L)**	39 ± 1 ^a^	50 ± 2 ^a,b^	47 ± 4 ^a,b^	54 ± 4 ^b^
**TC (mg/dL)**	57 ± 2 ^a^	60 ± 3 ^a,b^	59 ± 3 ^a,b^	68 ± 3 ^b^
**TG (mg/dL)**	81 ± 3 ^a^	92 ± 2 ^b^	89 ± 3 ^a,b^	104 ± 3 ^c^
**BUN (g/dL)**	8.5 ± 0.3 ^a^	11.4 ± 0.8 ^b^	9.6 ± 0.6 ^a,b^	11.4 ± 0.2 ^b^
**Creatinine (mg/dL)**	0.23 ± 0.00 ^a^	0.28 ± 0.01 ^b^	0.25 ± 0.01 ^a,b^	0.24 ± 0.01 ^a^
**ALB (g/dL)**	3.3 ± 0.0 ^b^	3.0 ± 0.1 ^a^	3.1 ± 0.0 ^a^	3.1 ± 0.0 ^a^
**TP (g/dL)**	5.3 ± 0.1 ^a^	5.1 ± 0.0 ^a^	5.2 ± 0.1 ^a^	5.3 ± 0.0 ^a^
**LDH (mg/dL)**	516 ± 20 ^a^	576 ± 22 ^a^	540 ± 27 ^a^	672 ± 87 ^a^

After 12 weeks of intervention, the biochemical indices were assessed at the end before mice were sacrificed. Data are presented as mean ± SEM, *n* = 8 mice/group. Different letters (a, b) in the same row indicate a significant difference at *p* < 0.05 determined using one-way ANOVA. AST, aspartate aminotransferase; ALT, alanine aminotransferase; TC, total cholesterol; TG, triacylglycerol; BUN, blood urea nitrogen; ALB, albumin; TP, total protein; LDH, lactate dehydrogenase.
